# Woven EndoBridge (WEB) Width at the Aneurysm Neck Level Affects Early Angiographic Aneurysm Occlusion

**DOI:** 10.1007/s00062-021-01034-0

**Published:** 2021-06-04

**Authors:** Marie Teresa Nawka, Gabriel Broocks, Rosalie McDonough, Jens Fiehler, Maxim Bester

**Affiliations:** Department of Neuroradiology, University Hospital Eppendorf, Hamburg, Germany

**Keywords:** Aneurysm, Brain, Vascular, Endovascular, Technique

## Abstract

**Purpose:**

Endovascular therapy with the Woven EndoBridge (WEB) device is a safe treatment approach, whereby neoendothelialization at the neck area is a crucial element for aneurysm occlusion. We hypothesized that WEB sizing at the aneurysmal neck level has an impact on early aneurysm occlusion.

**Methods:**

Patients with short-term follow-up digital subtraction angiography following WEB treatment of unruptured aneurysms were included. Aneurysms were categorized according to the Bicêtre Occlusion Scale Score (BOSS) as adequately (BOSS 0, 0′, 1) or partially occluded (BOSS 2, 3, 1 + 3). The WEB device dimensions, including the average aneurysm diameter (AADi) and the average neck diameter (ANDi) as well as baseline patient characteristics were documented.

**Results:**

In this study 75 patients with 76 aneurysms were included and 65 aneurysms showed adequate occlusion at short-term follow-up (86%). In univariable logistic regression analysis, smaller differences in WEB size to ANDi (D-ANDi) were significantly associated with adequate aneurysm occlusion (odds ratio, OR = 0.41, 95% confidence interval, CI 0.23–0.71, *p* = 0.002). Receiver operating characteristic (ROC) curve analyses displayed higher discriminative power for the D‑ANDi (AUC = 0.77, 95% CI 0.66–0.86, cut-off ≤2.9 mm) compared to the difference in WEB size to the average aneurysm diameter (D-AADi, AUC = 0.65, 95% CI 0.53–0.75, cut-off ≤1.0 mm).

**Conclusion:**

Smaller differences between the WEB width and ANDi were associated with adequate early aneurysm occlusion and might thus have a higher impact on the results than the traditional device sizing considering the mean aneurysm diameter. D‑ANDi ≤2.9 mm served as an optimal cut-off to classify occlusion after WEB treatment at the short-term follow-up. Further external validation is warranted.

**Supplementary Information:**

The online version of this article (10.1007/s00062-021-01034-0) contains supplementary material, which is available to authorized users.

## Introduction

The Woven EndoBridge system (WEB; MicroVention, Tustin, CA, USA) has been shown to be a valuable treatment option for both unruptured and ruptured wide-necked bifurcation (WNBA) intracranial aneurysms (IA) [1]. The cumulative 2‑year anatomical results of three good clinical practice (GCP) studies on the WEB device (WEBCAST, WEBCAST 2, and French Observatory) showed neck and aneurysm remnants in 29.8% and 19.0% of cases, respectively [1]. The combined study population for efficacy of the 3 trials included 113 unruptured aneurysms (93.4%) and 8 ruptured aneurysms (6.6%) [1]. In most other previous WEB analyses, unruptured aneurysms represented the majority of the patient collective [2–5]. To uniformly categorize IA occlusion rates following WEB implantation, various occlusion scores, including the WEB occlusion scale (WOS) and the newer Bicêtre occlusion scale score (BOSS), have been developed and incorporated into the clinical routine [6–8]. Incomplete IA occlusion (e.g., the presence of neck and aneurysm remnants) is associated with a higher risk of re-rupture and should therefore be avoided when possible [9]. Previous studies have assessed the ability to predict occlusion outcome following coil embolization and flow diversion based on certain aneurysm metrics [10, 11]. Other investigations of WEB-based treatment strategies have shown aneurysm size, neck diameter, and dome-neck ratio to be associated with aneurysm occlusion [4, 12, 13]. Correct WEB sizing, therefore, is a crucial component of the initial treatment process, and is likely important for final WEB shape modification and long-term aneurysm occlusion [14]. Nevertheless, there remains no universal approach to appropriate WEB size selection. The WEB device selection guide (MicroVention) provides instructions to this end. Only measurements of the aneurysm width and height are necessary to select the appropriate WEB shape (WEB SL or SLS). These parameters are determined by measuring the average aneurysm width and the smallest aneurysm height from two different projections. Despite excellent treatment results applying the aforementioned device selection rules, sizing of the device at the aneurysm neck level appears to be crucial. The braided dense mesh surface of the WEB device serves as an interface between the aneurysm neck and the parent vessel, while the neck area represents a barrier to the inflow of blood [6]. This in turn leads to neointimal coverage of the WEB device in the direction of the parent artery, resulting in aneurysm thrombosis and eventually aneurysm occlusion [6, 15, 16].

The WEB devices of varying sizes are intended to adapt to a range of aneurysmal widths and slight changes in the WEB structure can occur following deployment [17]. In addition, aneurysmal width and the neck diameters can vary and discrepancies between the WEB size and the mean neck width can potentially influence flow disruption and therefore neointimal coverage at the neck area.

We hypothesized that WEB sizing in relation to the average aneurysm neck diameter (ANDi) is associated with aneurysm occlusion results at the 6‑month follow-up examination. The findings of this analysis could further aid neurointerventionalists in the selection of appropriate device size and thus optimize occlusion rates of unruptured aneurysms in the future.

## Material and Methods

### Study Design

This represents a single-center, retrospective study of consecutively screened patients with unruptured IA treated with the WEB device in our neuroendovascular center between February 2014 and December 2019 (*n* = 93). Only anonymized data were analyzed after ethics review board approval, and the local ethics committee waived informed consent after review. Of the patients seven were lost to follow-up. Of the 86 remaining patients, 11 patients did not undergo routine digital subtraction angiography (DSA) 6 months post-treatment but were present for subsequent follow-up appointments. In addition to DSA, regular magnetic resonance imaging (MRI) follow-up examinations at 6 and 18 months after WEB treatment are routinely performed at our center as well as longer-term MRI examinations with both non-contrast and contrast-enhanced MRA. These studies were collected, if available.

### Interventional Procedure

All endovascular treatments were performed by senior interventional neuroradiologists using a biplane angiographic system (Allura Clarity FD 20/20; Philips Healthcare, Best, The Netherlands). Following transfemoral catheterization, a selective series of images in anteroposterior and lateral projections, a three-dimensional rotational angiography and a working view projection were captured. The size of the selected WEB device was chosen based on measurements of aneurysm width and height on two-dimensional DSA images. Implant sizes were selected according to the manufacturer’s instructions for use. If not satisfactorily positioned, the WEB device was recaptured and repositioned. Definitive WEB placement was confirmed by a final DSA run following detachment.

Dual antiplatelet medication was started 7 days prior to treatment, consisting of a daily dose of 100 mg ASA and 75 mg clopidogrel. Periprocedural antiaggregation treatment was conducted with 3000 IU of heparin. If no adjunctive device was placed, antiaggregation with 100 mg of ASA was continued for 6 weeks following the procedure. If additional stenting of the IA was performed, follow-up antiaggregation treatment consisting of 75 mg clopidogrel daily for 3 months and lifelong ASA (100 mg) was prescribed.

### Image Evaluation

Our standard follow-up regimen includes both MRI and DSA at 6 months, followed by an additional MRI control after 18 months. Short-term aneurysm occlusion rates were assessed with the BOSS grading scale by an experienced senior neuroradiologist with >12 years of neurointerventional practice (M.B.) [8]. Blinded image ratings were conducted to ensure consistency of image evaluation. Aneurysm occlusion was graded as follows: 0 represents complete occlusion, 0′ shows opacification of the proximal recess, 1 indicates intradevice filling, 2 denotes a neck remnant, 3 signifies an aneurysm remnant and 1 + 3 specifies contrast agent inside and around the device [8]. According to Caroff et al. the scores 0 and 0′ are regarded as complete occlusion [8]. In cases with isolated residual intra-WEB filling (BOSS 1), complete occlusion is often observed at subsequent follow-up examinations [8, 18]. Hence, we categorized BOSS scores 0, 0′ and 1 as adequately occluded aneurysms, whereas BOSS scores 2, 3 and 1 + 3 were considered to be incompletely occluded [8]. Clinical outcome was assessed according to the modified Rankin scale score (mRS) after 6 months.

### Aneurysm Morphometrics

**Fig. 1:**
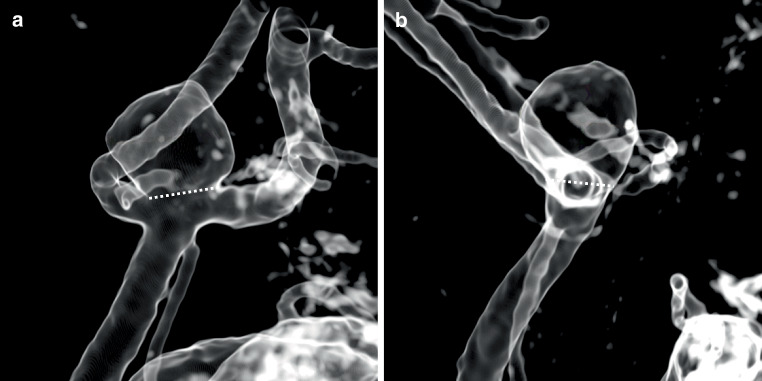
Measurement of the average neck diameter of an AcomA aneurysm. Aneurysmal neck width determination from WEB sizing *view* ***a****,* as illustrated by the dotted line. *Neck diameter a* was 3.4 mm (**a**). Aneurysmal neck width determination from *orthogonal* WEB sizing *view* ***b****,* as illustrated by the dotted line. *Neck diameter* *b* was 4.0 mm. The mean neck diameter of the measurements *a *and *b *was then calculated to be 3.7 mm (**b**)

Aneurysm morphology was analyzed using 3D rotational angiography (3D RA). Aneurysm width and neck width measurements were identified following reconstruction of orthogonal WEB sizing projections on a dedicated 3D workstation. Fig. 1 exemplifies the neck measurement of an AcomA aneurysm. Discrepancies between each WEB diameter and the corresponding aneurysm/neck diameter were noted. Dome-to-neck ratio was defined as the average dome width relative to the average neck diameter [19]. All measurements are expressed in millimeters (mm).

### WEB Sizing

Using the manufacturer’s WEB sizing scale, the average aneurysm diameter range determines the choice of the WEB size. All cases were categorized into one of the following groups, regardless of WEB type (WEB SL/SLS): 1) the selected WEB size is within the indicated range, 2) the selected WEB size matches the next smaller size (undersizing), or 3) the selected WEB size matches the next larger size (oversizing). If, for example, the average aneurysm width was found to be 5.2 mm, the WEB sizing scale recommends a WEB width of 6 mm (WEB SL). In this case, selection of a WEB SL 6 × 3 or 6 × 4 (depending on the height of the aneurysm) would be defined as group 1 (within range). A WEB SL 5 × 2 or 5 × 3 would be labelled as group 2 (next smaller size) and a WEB SL 7 × 3, 7 × 4, or 7 × 5 would represent the next larger size and therefore fall into group 3.

### Statistical Analysis

Absolute and relative frequencies for all patient characteristics are presented separately for adequately and incompletely occluded aneurysms (binarized at a BOSS score ≤1/>1). To test for normal distribution of the data, we performed the Shapiro-Wilk test. Categorical variables were compared applying the χ^2^-test and are described as number and percentage. For metric variables, the Mann-Whitney U‑test was performed and values are presented as median ± interquartile range (IQR). Statistics were performed for the following patient and aneurysm parameters: age, sex, aneurysm location, mRS, WEB type, WEB width, average aneurysm diameter, average neck diameter, D‑AADi, D‑ANDi, dome-neck-ratio and WEB sizing groups 1–3. We used univariable logistic regression analysis to evaluate morphological aneurysm characteristics individually and compared adequately occluded aneurysms with incompletely occluded aneurysms. Additional ordinal regression analysis was performed to assess the probability for aneurysm occlusion per BOSS grade according to D‑ANDi. Univariable receiver operating characteristic (ROC) curves were used to evaluate the ability of the D‑AADi and the D‑ANDi to discriminate between adequately and incompletely occluded aneurysms. Thresholds for optimal sensitivity and specificity were also calculated with the Youden index, defined as the maximum of sensitivity plus the specificity minus one [20, 21]. A *p*-value of <0.05 was considered statistically significant. Analyses were conducted using MedCalc Statistical Software version 19.1.7 (MedCalc Software bv, Ostend, Belgium).

## Results

### Patient Characteristics and Aneurysm Morphometrics

A total of 75 patients with 76 intracranial aneurysms were included in this study, 63 (84%) patients were female and 12 (16%) were male, with a mean age of 56 ± 10 years (range 26–78 years) at time of treatment. Median follow-up time for short-term DSA was 190 days (i.e., 6.2 months; range 112–137 days) and 574 days (i.e., 18.9 months; range 488–803 days) for long-term MRA. Of the aneurysms 60 were treated with a WEB SL whereas a WEB SLS was placed in 16 cases, 49 aneurysms were treated with the WEB 21/27/33 system and the WEB 17 system was used to treat 27 aneurysms. Of the aneurysms 46 were located in the anterior circulation and 30 aneurysms were located in the posterior circulation. A detailed overview is given in Table 1.

**Table 1:** Distribution of aneurysm location

Site	No. of aneurysms	% of total
*Anterior circulation*		
Anterior communicating artery	22	29.0
Anterior choroidal artery	1	1.5
Middle cerebral artery	9	9.0
Internal carotid artery	16	21.0
*Posterior circulation*		
Basilar tip	24	31.6
Posterior communicating artery	2	2.6
Posterior inferior cerebellar artery	4	5.3

In 3 patients an additional stent was placed. In 71 patients mRS at 6 months was 0 and 3 patients showed an mRS of 1 and 2 patients had an mRS of 2.

At the short-term follow-up, overall adequate aneurysm occlusion was achieved in 65 cases (BOSS 0 = 27, BOSS 0′ = 24, BOSS 1 = 14), neck remnants (BOSS 2) were detected in 7 cases and 4 cases were classified as aneurysm remnants (BOSS 3 = 1, BOSS 1 + 3 = 3). Of the aneurysm remnants (BOSS 3) 3 were retreated with coiling (*n* = 2) or stent-assisted coiling (*n* = 1) within 1 year after the initial procedure.

Long-term follow-up MRA was performed in 36 cases (47%), showing complete aneurysm occlusion (BOSS 0, 0′) in 30 cases (83%) or neck remnants (BOSS 2) in 6 aneurysms (17%). No aneurysm remnants were identified. The BOSS 1 gradings could not be assessed as intradevice filling is not visible on MRI [22].

Table 2 provides an overview of the overall patient collective, stratified by adequate (BOSS 0, 0′, 1) and incomplete (BOSS 2, 3, 1 + 3) aneurysm occlusion at the short-term follow-up [8].

**Table 2:** Demographic information and aneurysm morphometrics, stratified by adequate and incomplete aneurysm occlusion at the 6‑month follow-up

Patient and aneurysm characteristics	Group 1 = adequate aneurysm occlusion (BOSS 0, 0′, 1)	Group 2 = incomplete aneurysm occlusion (BOSS 2, 3, 1 + 3)	Group comparison *p*-value
Subjects, *n* (%)	65 (86)	11 (14)	–
Age (years), median (IQR^a^)	55 (51–61)	60 (48–66)	0.535
Female sex, *n* (%)	54 (83)	9 (82)	0.741
Anterior aneurysm location, *n* (%)	41 (63)	6 (55)	0.839
mRS 6 months, median (IQR^a^)	0 (0–0)	0 (0–0)	0.122
WEB SL, *n* (%)	51 (78)	9 (82)	0.883
WEB width (mm), median (IQR^a^)	6 (4.5–7)	7 (6–8.8)	0.012*
Average aneurysm diameter (mm), median (IQR^a^)	4.9 (3.8–5.8)	5.5 (5.0–8.0)	0.049*
Average neck diameter (mm), median (IQR^a^)	3.8 (2.9–4.4)	3.6 (3.3–4.5)	0.647
D‑AADi (mm), median (IQR^a^)	0.9 (0.7–1.2)	1.1 (0.8–1.4)	0.126
D‑ANDi (mm), median (IQR^a^)	2.0 (1.2–2.8)	3.7 (2.5–4.9)	0.004*
Dome-neck-ratio (average), median (IQR^a^)	1.3 (1.1–1.6)	1.6 (1.4–1.9)	0.042*
WEB sizing (group 1), *n* (%)	42 (65)	6 (55)	0.762
WEB sizing (group 2), *n* (%)	7 (11)	0 (0)	0.563
WEB sizing (group 3), *n* (%)	16 (25)	5 (45)	0.287

*Significant parameters in group comparison

^a^*IQR* interquartile range

### Univariable Logistic Regression and Ordinal Regression Analyses

We used univariable logistic regression analysis to assess predefined aneurysm morphometrics individually and evaluated their impact on aneurysm occlusion. The variables WEB width (odds ratio, OR = 0.60, 95% confidence interval, CI 0.41–0.88, *p* = 0.008), average aneurysm diameter (OR = 0.62, 95% CI 0.42–0.92, *p* = 0.017), D‑ANDi (OR = 0.41, 95% CI 0.23–0.71, *p* = 0.002), and dome-neck-ratio (OR = 0.12, 95% CI 0.02–0.86, *p* = 0.035) were significantly associated with adequate aneurysm occlusion.

**Fig. 2:**
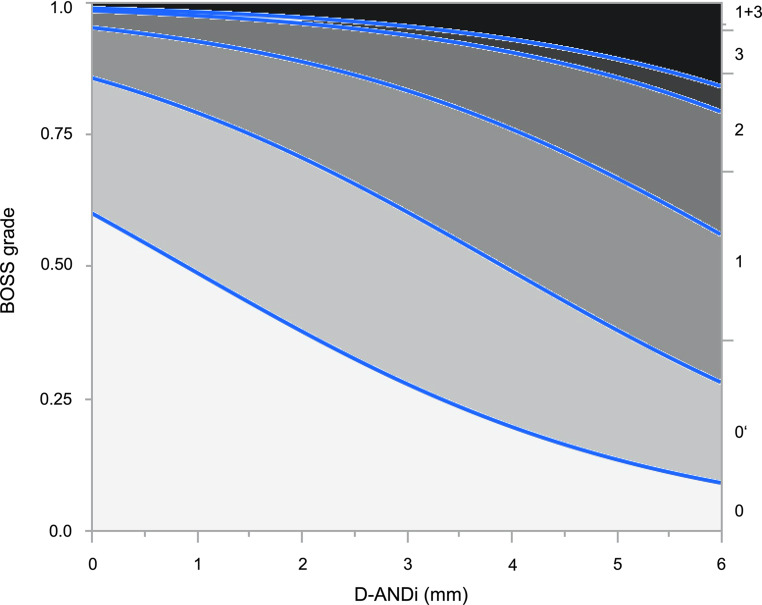
Probability of each BOSS grade at the short-term follow-up in accordance with D‑ANDi. The diagram shows the probability curves for each BOSS grade (*y‑axis*) per D‑ANDi (*x‑axis*). The blue lines divide the probabilities for the six different BOSS outcomes into six areas, one area per BOSS grade. The size of each area decreases with an ascending BOSS grade, illustrating a higher probability for incomplete aneurysm occlusion (=higher BOSS grade) if D‑ANDi increases

Because the six different BOSS outcomes can be graded in an ascending order, we performed additional ordinal regression analyses. The parameters D‑AADi and D‑ANDi were tested to assess their respective impact on the probability for aneurysm occlusion per BOSS grade (Table 3). Fig. 2 illustrates the probability for each BOSS grade according to D‑ANDi.

**Table 3:** Ordinal regression analysis to predict aneurysm occlusion per BOSS grade

Aneurysm parameter	OR	95% CI	*p*-value
D‑AADi (mm)	1.85	0.78–4.36	0.161
D‑ANDi (mm)	1.65	1.15–2.38	0.007

*D‑ANDi* Difference in WEB size to average neck width

### Receiver Operating Characteristic (ROC) Curve Analysis

To assess the ability of D‑AADi and D‑ANDi to predict occlusion rates as determined by early follow-up DSA, we performed univariable ROC curve analyses. Besides D‑ANDi and D‑AADi, the variables WEB width and the dome-neck ratio were included in the ROC analysis, because they were significantly associated with adequate aneurysm occlusion in the univariable regression analysis. The ability to discriminate between adequate and incomplete aneurysm occlusion was higher for D‑ANDi (AUC = 0.77, 95% CI 0.66–0.86), compared to D‑AADi (AUC = 0.65, 95% CI 0.53–0.75). The sensitivity and specificity for D‑ANDi were 80% and 73%, respectively, using ≤2.9 mm as an optimal cut-off. Table 4 lists the respective AUC (with 95% confidence intervals), sensitivity, specificity, Youden index, and associated criterion values for all ROC curves.

**Table 4:** ROC curve analysis of D‑ANDI, D‑AADi, WEB width and dome-neck-ratio, which discriminate between adequately and incompletely occluded aneurysms at the short-term follow-up

Aneurysm parameter	AUC	95% CI	Sensitivity	Specificity	Youden index	Associated criterion
D‑ANDi (mm)	0.77	0.66–0.86	80	73	0.5	≤2.9
WEB width (mm)	0.73	0.62–0.83	71	64	0.3	≤6.0
Dome-neck ratio (average)	0.65	0.58–0.79	68	73	0.4	≤1.4
D‑AADi (mm)	0.65	0.53–0.75	68	64	0.3	≤1.0

*D‑ANDi* Difference in WEB size to average neck width

**Fig. 3:**
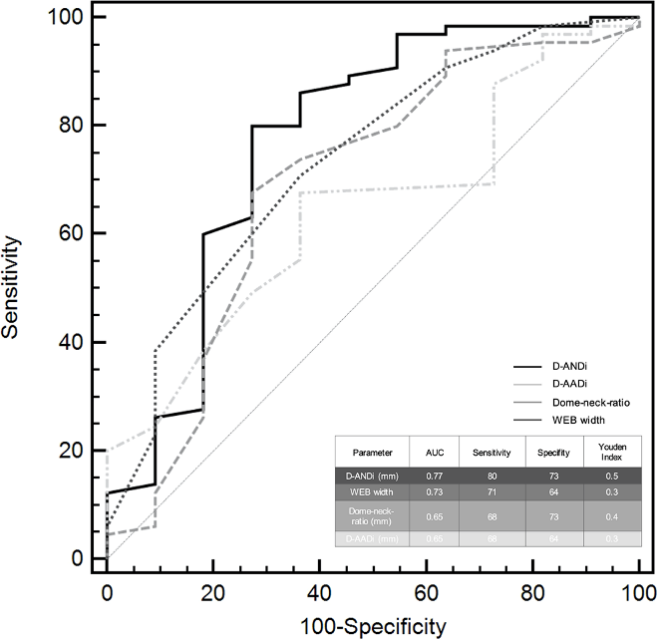
Univariable ROC curve analysis for D‑AADi and D‑ANDi. Solid lines represent univariable ROC curve analysis of differences between D‑ANDI, D‑AADi, WEB width and dome-neck ratio by aneurysm occlusion based on the BOSS scale. The ability to discriminate between adequate and incomplete aneurysm occlusion by AUC was highest for D‑ANDi. The delicate line along the middle of the diagram is the null predictor

Fig. 3 illustrates the univariable ROC analysis for D‑ANDI, D‑AADi, WEB width and dome-neck-ratio evaluated independently.

To illustrate the value of the D‑ANDi on short-term aneurysm occlusion, supplementary Fig. 1 depicts four different cases with respect to the different BOSS outcomes. Two WEB-SL cases and two WEB-SLS cases are presented, each with a D-ANDi of >2.9 mm and of <2.9 mm, respectively.

## Discussion

Flow disruption of wide-necked bifurcation aneurysms with the WEB device is an innovative endovascular treatment approach, with demonstrated satisfactory mid-term and long-term adequate occlusion rates in various studies [1, 13, 14, 23]. Most of the published series included unruptured aneurysms, and the corresponding clinical results showed good safety profiles [2, 3, 5]. While the ability of different aneurysm characteristics to serve as predictive factors for adequate aneurysm occlusion has been investigated, it is currently unclear which discriminators are mainly responsible for aneurysm recanalization or recurrence [4, 12–14, 24]. To the best of our knowledge, none of the aforementioned studies focused on the sizing of the WEB device according to the mean aneurysm neck diameter and its potential predictive value for aneurysm occlusion. As flow disruption/diversion at the aneurysmal neck surface is regarded as being a crucial factor for aneurysm occlusion after WEB treatment [6, 15], we aimed to evaluate the impact of major deviations between the WEB size and aneurysm/neck width and to assess their influence on aneurysm occlusion at the short-term follow-up.

In our study the rates of adequate (BOSS 0, 0′, 1) and incomplete (BOSS 2, 3, 1 + 3) aneurysm occlusion on early angiographic images were 86% and 14%, respectively. These numbers are in line with those from the literature as aneurysm occlusion rates following WEB treatment have been shown to be generally satisfactory and are indeed considered to be superior to coiling or stent-assisted coiling in similar patient cohorts [12, 23, 25–28].

The importance of correct WEB sizing in the context of aneurysm occlusion has been established; slight oversizing of the device with respect to the mean aneurysm width is recommended and is likely crucial to avoid incomplete occlusion [4, 17, 29]. In our study, we did not find an association between different sizing strategies (groups 1, 2, or 3) and adequate aneurysm occlusion. Nevertheless, undersizing could lead to aneurysm recurrence and is therefore likely unfavorable [4, 29]. With the aim of predicting early occlusion rates in aneurysms treated with correctly sized WEB devices using the established workflow, we measured aneurysm and neck diameters in orthogonal planes and calculated the mean values. This approach seems to be superior to basing WEB size selection on a single measurement of length because the correct choice of the WEB width is highly dependent on the average aneurysm diameter. The selected WEB size is usually chosen with respect to the mean aneurysm width, while rarely being exactly equal in value. We therefore identified differences between each WEB width and the average aneurysm and neck diameter, respectively. The official WEB sizing scale is limited in its applicability to all aneurysms as each WEB size has been developed to fit a range of aneurysmal widths. This in turn could lead to unpredictability of device behavior, particularly in respect to possible shape modification over time [17]. Adequate wall apposition can be achieved if the WEB is slightly oversized, whereas excessive oversizing might result in an unbalanced neck coverage, potentially leading to an unfavorable crescent shape of the device [30]. If the neck-vessel border is sufficiently covered by the wire mesh, intra-aneurysmal thrombosis following blood flow disruption and endothelial cell growth at the aneurysmal neck are induced, thereby initiating aneurysm occlusion [15]. Considering the importance of precise aneurysm measurements, we hypothesized that flow diversion and subsequent neck endothelialization is affected by discrepancies between the WEB size and the mean neck diameter and could thus potentially serve as a predictor of early aneurysm occlusion. We observed a greater discriminative power for the parameter D‑ANDi compared to D‑AADi to reliably predict early occlusion rates in unruptured WEB treated aneurysms, independent of general WEB sizing (as proposed by the official sizing chart), thereby confirming our hypothesis. In the treatment setting of unruptured aneurysms, the operator has more time to prepare the procedure and choice of the WEB device might be less stressful than in the setting of an acutely ruptured aneurysm with the primary objective of fast aneurysm occlusion. Hence, adequate comparison of the outcome for patients with unruptured and ruptured aneurysms after WEB treatment might be impeded due to great variations in patient characteristics (e.g. aneurysm type, location, comorbidities) [4, 31].

In addition, conventional WEB sizing strategies did not affect aneurysmal occlusion rates at the short-term follow-up, suggesting that exact WEB sizing based on sole consideration of the average aneurysm size is potentially less important than previously assumed. As the definition of the mean aneurysmal width as a standalone parameter might therefore be insufficient, a 3D adjustment of the WEB device to effectively cover the neck area and simultaneously adhere to the aneurysmal wall by completely filling its width might be beneficial for complete and stable aneurysm occlusion. Despite the recommendations of rather oversizing the device, it should be noted that, especially in cases of irregularly shaped aneurysms, it might sometimes be difficult to strictly adjust the WEB width to the neck size. Cagnazzo et al. found that the aneurysm shape independently influenced aneurysm occlusion at follow-up angiograms and that the presence of a taller aneurysm dome was associated with increased WEB shape modification [13]. A compression or modification of the WEB device has already been reported to be potentially associated with aneurysm recanalization [32]; however, this appears to be independent of undersizing or oversizing the device [14]. Major discrepancies between the WEB size and the corresponding neck size could nevertheless cause a change in WEB shape, thus affecting its mesh configuration and potentially influencing its porosity. This could in turn facilitate continued blood flow into the WEB device and the aneurysm, thereby hindering complete occlusion.

The importance of the aneurysmal dome and neck size and their impact on aneurysm occlusion have been investigated for other endovascular treatment options. An early study of risk factors for partial occlusion following coil embolization included an analysis of aneurysm and neck diameters and showed that large aneurysmal widths are predictive of incomplete immediate aneurysm occlusion [11] but not for recanalization delay [33]. Nakazaki et al. found that the median neck diameter of aneurysms treated with stent-assisted coiling was predictive for progressive occlusion [34]. A new parameter suggested to be predictive of aneurysm occlusion after flow diverter treatment was discussed by Paliwal et al. in 2019 [10]. Here, they calculated the aneurysm ostium surface area to the parent artery diameter ratio and found that high ostium and neck ratios could predict incomplete occlusion after flow diversion [10].

## Limitations

This study has several limitations. First, our analysis was performed on a relatively small retrospective single-center data set including only unruptured aneurysms. Image evaluation was accomplished by a single reader. Second, we only evaluated short-term angiographic aneurysm occlusion rates of unruptured aneurysms. Third, occlusion outcomes might be also dependent on the respective operator and the clinical experience in WEB treatment, especially in respect to aneurysm measurements and appropriate choice of the device. Despite the reliable performance of D‑ANDi in this single-center study, external validation to confirm the value of this parameter in a broad range of clinical settings is necessary. Future studies using a 3D simulation software and incorporating complex aneurysm shapes into the device selection process could facilitate the understanding of the interaction between the WEB device and certain aneurysm characteristics.

## Conclusion

While WEB size selection is dependent on the average aneurysm width, oversizing might not be as crucial as previously assumed. Moreover, determination of the ANDi appears to be beneficial as our analyses demonstrated that the D‑ANDi was associated with adequate aneurysm occlusion at the short-term follow-up and may thus help in the identification of aneurysms at risk for incomplete occlusion at the short-term follow-up. Further external validation is necessary.

## Supplementary Information

Supplementary Fig. 1

Caption supplementary Fig. 1

## Abbreviations

AcomA: Anterior communicating artery
ASA: Acetylsalicylic acid
AUC: Area under the curve
BOSS: Bicêtre occlusion scale score
D‑AADi: Difference in WEB size to average aneurysm width
D‑ANDi: Difference in WEB size to average neck width
mRS: Modified Rankin scale
ROC: Receiver operating characteristics
WNBA: Wide-necked bifurcation aneurysm
WOS: Web occlusion scale
3D RA: 3D rotational angiography
