# Supplementary figures and images for: Woven EndoBridge (WEB) Width at the Aneurysm Neck Level Affects Early Angiographic Aneurysm Occlusion
Source: Clin Neuroradiol. 2021 Jun 4;32(1):89–97. doi: 10.1007/s00062-021-01034-0 (PMC8894173; doi:10.1007/s00062-021-01034-0)

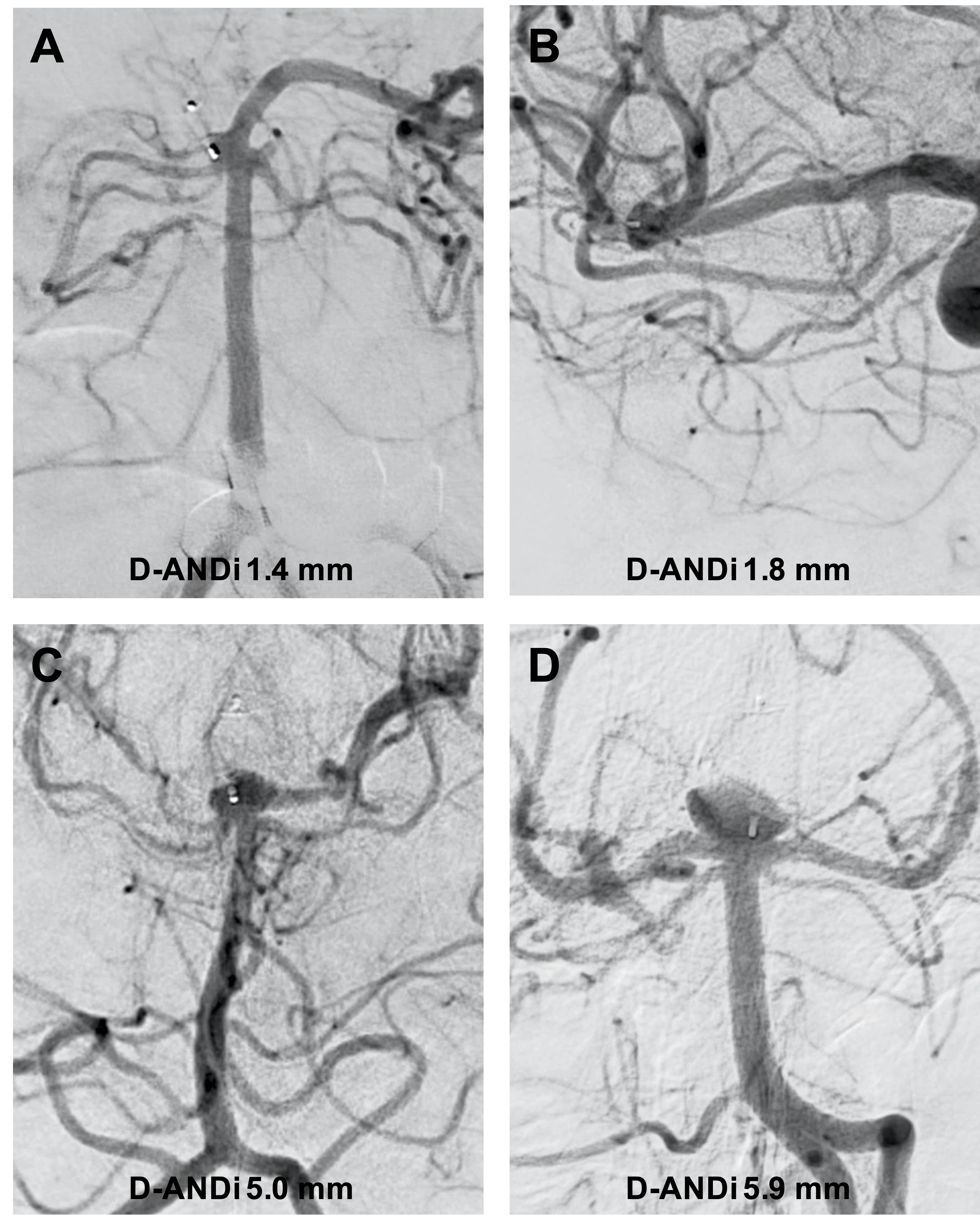

Supplement: Supplementary file 1 — Supplementary Fig. 1 [file 62_2021_1034_MOESM1_ESM.tiff]
