# Supplementary material for: Woven EndoBridge (WEB) Width at the Aneurysm Neck Level Affects Early Angiographic Aneurysm Occlusion
Source: Clin Neuroradiol. 2021 Jun 4;32(1):89–97. doi: 10.1007/s00062-021-01034-0 (PMC8894173; doi:10.1007/s00062-021-01034-0)
Supplement: Supplementary file 2 — Caption supplementary Fig. 1 [file 62_2021_1034_MOESM2_ESM.docx]

**Supplemental fig. 1. Illustration of the value of the D-ANDi on short-term aneurysm occlusion.** (A) Short-term follow-up DSA after treatment of a basilar tip aneurysm with a WEB-SL 4x3. The D-ANDi was 1.4 mm, the BOSS grade was 0. (B) Short-term follow-up DSA after treatment of a MCA aneurysm with a WEB-SLS 6. The D-ANDi was 1.8 mm, the BOSS grade was 0’. (C) Short-term follow-up DSA after treatment of a basilar tip aneurysm with a WEB-SL 9x6. The D-ANDi was 5.0 mm, the BOSS grade was 2. (D) Short-term follow-up DSA after treatment of a basilar tip aneurysm with a WEB-SLS 11. The D-ANDi was 5.9 mm, the BOSS grade was 1+3.
